# Genome-Scale Metabolic Model Analysis of Metabolic Differences between Lauren Diffuse and Intestinal Subtypes in Gastric Cancer

**DOI:** 10.3390/cancers14092340

**Published:** 2022-05-09

**Authors:** Seungyoon Nam, Yongmin Lee

**Affiliations:** 1 Department of Health Sciences and Technology, Gachon Advanced Institute for Health Sciences and Technology (GAIHST), Gachon University, Incheon 21999, Korea; dydrkf432@gachon.ac.kr; 2 Department of Genome Medicine and Science, AI Convergence Center for Medical Science, Gachon Institute of Genome Medicine and Science, Gachon University Gil Medical Center, Gachon University College of Medicine, Incheon 21565, Korea

**Keywords:** genome-scale metabolic model, transcriptome, metabolism, gastric cancer

## Abstract

**Simple Summary:**

Gastric cancer (GC) is one of the most deadly cancers globally. GC is a heterogeneous cancer type and has different histological subtypes. The aim of our study is to identify the metabolic differences between the subtypes, which will lead to a better understanding of metabolism in GC heterogeneity.

**Abstract:**

Gastric cancer (GC) is one of the most lethal cancers worldwide; it has a high mortality rate, particularly in East Asia. Recently, genetic events (e.g., mutations and copy number alterations) and molecular signaling associated with histologically different GC subtypes (diffuse and intestinal) have been elucidated. However, metabolic differences among the histological GC subtypes have not been studied systematically. In this study, we utilized transcriptome-based genome-scale metabolic models (GEMs) to identify differential metabolic pathways between Lauren diffuse and intestinal subtypes. We found that diverse metabolic pathways, including cholesterol homeostasis, xenobiotic metabolism, fatty acid metabolism, the MTORC1 pathway, and glycolysis, were dysregulated between the diffuse and intestinal subtypes. Our study provides an overview of the metabolic differences between the two subtypes, possibly leading to an understanding of metabolism in GC heterogeneity.

## 1. Introduction

Gastric cancer (GC) is one of the most common cancers worldwide and ranks second among cancer-related deaths [1,2]. Recent advances in cancer diagnosis and treatment have resulted in limited improvements in GC-related mortality [3], and estimates suggest that gastric cancer–related mortality will continue to increase [4]. To understand the molecular basis of GC, several studies have elucidated the genetic landscapes and oncogenic signaling pathways of GC and identified biomarkers predicting prognosis and response to treatment [5,6]. In addition, GC has different histological subtypes according to the Lauren classification, namely, diffuse and intestinal [1]. The two subtypes have different clinical and molecular characteristics, including etiology and prognosis [2]. The intestinal type is associated with *Helicobacter pylori* (*H. pylori*) infection, whereas the diffuse type is more common in women and younger patients [3]. In addition, the expression of human epidermal growth factor receptor-2 (HER2) is more prevalent in patients with the intestinal subtype, with better outcomes than in patients with the diffuse subtype [2]. Thus, trastuzumab plus chemotherapy is the standard first-line therapy for HER2-positive advanced or esophagogastric junction cancers [4].

Common oncogenic signaling pathways are involved in the tumorigenesis of GC: the Hippo pathway, WNT pathway, Hedgehog, TGFβ signaling, cell adhesion, and chromatin remodeling [5]. Recently, metabolism in cancer has attracted attention since the reprogramming of cellular metabolism is both a direct and indirect consequence of oncogenic mutations [6]. To investigate metabolism using transcriptomics, genome-scale metabolic models (GEMs) have been applied to cancer [7,8,9,10,11]. Gene–protein–reaction (GPR) annotations, a set of rules that define the isoenzymes or protein complexes that catalyze each reaction, enable the mapping of transcriptomic or proteomic measurements to GEM reactions [9]. However, despite the importance of histological differences in GC, metabolic differences between the GC subtypes have not been investigated, particularly using transcriptomics-based GEMs.

To address this limitation, we applied transcriptome-based GEMs to a stomach adenocarcinoma dataset from the Cancer Genome Atlas (TCGA-STAD) [12] using iMAT [13] and Metabolizer [14]. This dataset includes patients with diffuse and intestinal GC subtypes. We further validated the differences in metabolic pathways between the two subtypes using independent transcriptome datasets and a metabolic profiling dataset.

## 2. Materials and Methods

### 2.1. GC Dataset

In our study, we downloaded a dataset of patients with GC, TCGA-STAD (version 2019-12-06) [12], curated by UCSC Xena [15]. The clinical information in TCGA-STAD was available for 580 GC samples, and 450 of them had gene expression profiles obtained by RNA-seq. A total of 185 patients with GC had available information on Lauren diffuse (*n* = 81) and intestinal (*n* = 104) subtypes. The rest of the patients (i.e., 450 minus 180) were not specified in terms of Lauren histologic subtypes and were excluded.

### 2.2. Hallmark Gene Set Analysis for Diffuse and Intestinal Subtypes and Principal Component Analysis (PCA)

For hallmark gene set analysis, we first calculated *t*-test statistics and false discovery rates (FDRs) of gene expression profiles of 1379 metabolic genes from the Kyoto Encyclopedia of Genes and Genomes (KEGG) [16] in Lauren diffuse versus intestinal subtype. Differentially expressed genes (DEGs) between the two Lauren GC subtypes were obtained using an FDR cutoff of 0.1. Subsequently, statistically significant hallmark gene sets were obtained using the function “Investigate gene sets” in MIT MSigDB [17].

For principal component analysis (PCA), the gene expression profiles of the 1379 metabolic genes were used. PCA was performed to determine the separation between the two Lauren subtypes.

### 2.3. iMAT Analysis of Diffuse and Intestinal GC Subtypes

We applied the iMAT [13] method implemented in the COBRA toolbox [18] to the TCGA-STAD dataset. Following the iMAT documentation [13], we applied the algorithm to each GC subtype using gene expression profiles as input. iMAT converted the numerical expression values into three categorical levels: low, intermediate, and high expression. For each gene, the boundary between intermediate and high expression was mean +0.3 × σ, and the boundary between intermediate and low expression was mean −0.3 × σ, where σ is the standard deviation of expression values for the gene [19]. iMAT uses Recon3D [20] as its default GEM. This analysis generated highly abundant metabolic reactions for each GC subtype. In addition, a subsystem, defined as a series of metabolic reactions [13] related to a specific metabolic process, is reported in this analysis. We compared highly abundant reactions between the two GC subtypes, obtaining common and exclusive reactions between the subtypes.

### 2.4. Metabolizer Analysis of Diffuse and Intestinal GC Subtypes

The gene expression profiles of the two subtypes were used as input in Metabolizer [14], which obtained activities for 96 modules. A module consisted of sequential metabolic reactions in a subpart of a KEGG metabolic pathway. Afterwards, hierarchical clustering was performed for the module activities.

### 2.5. Validation of Significant Metabolic Pathways in Other GC Datasets and a Metabolic Profiling Dataset

To validate the gene of the modules reported by Metabolizer, we obtained two independent preprocessed datasets of patients with GC from the Gene Expression Omnibus (GEO) [21]: GSE15459 (*n* = 45 for diffuse subtype; *n* = 50 for intestinal subtype) [22] and GSE47007 (*n* = 12 for diffuse subtype; *n* = 18 for intestinal subtype) [23]. In each dataset, we calculated the logarithms (base 2) of fold changes (log2(FC)) in gene expression in the diffuse subtype compared to the intestinal subtype. Then, we generated a heat map of the log2(FC) of the genes.

In addition, to inspect the gene modules in terms of GC cell models, we reanalyzed gene expression profiles of the modules’ genes in diffuse (MKN1, KATOIII, and MKN45) and intestinal (MKN7 and NCI-N87) GC cell lines [24,25] from a publicly available resource, the Genomics of Drug Sensitivity in Cancer (GDSC) [26]. Subsequently, we compared the gene expression profiles of the modules’ genes between the TCGA dataset and GC cell lines.

For experiments on metabolites, we reanalyzed lipid metabolic profiles of diffuse and intestinal GC cell lines. A publicly available repository, the Dependency Map (DepMap) portal [27], has the Cancer Cell Line Encyclopedia (CCLE) database (version CCLE2019) [28], including metabolomic profiling of 124 polar and 101 lipid species in 1826 cancer cell lines using hydrophilic interaction chromatography and reversed-phase chromatography [28]. In the database, eight diffuse (FU97, MKN1, NUGC4, OCUM1, SNU601, SNU668, KATOIII, and MKN45) and six intestinal (SH10TC, HUG1N, NCIN87, SNU719, SNU216, and MKN7) GC cell lines [24,25] were available. Subsequently, we identified differential metabolites between diffuse and intestinal GC cell lines by using *T* tests.

## 3. Results

### 3.1. Overview

**Figure 1:**
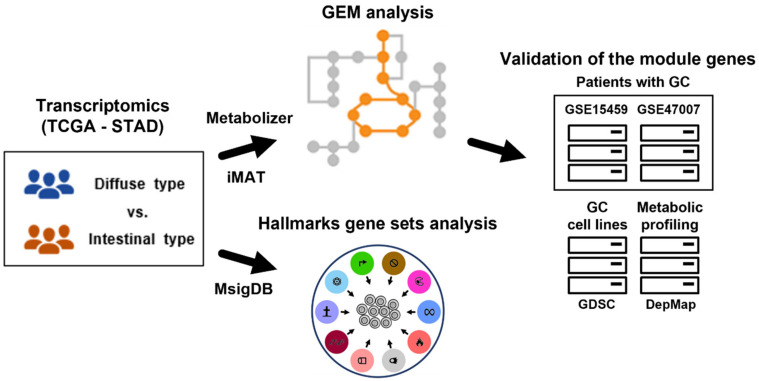
Overview of this study. Gene expression profiles of Lauren diffuse and intestinal GC subtypes were used to extract subsets of GEMs (i.e., all metabolic reactions in a cell) using iMAT and Metabolizer. The genes in GEM modules were validated in independent GC datasets. To identify functional contexts, gene set analysis for hallmark gene sets from MSigDB was applied to the gene expression profiles. GC: gastric cancer; TCGA: the Cancer Genome Atlas; STAD: stomach adenocarcinoma; GEM: genome-scale metabolic model; iMAT: integrative metabolic analysis tool; MSigDB: the Molecular Signatures Database; GDSC: the Genomics of Drug Sensitivity in Cancer; and DepMap: the Dependency Map.

We obtained differential gene expression profiles between diffuse and intestinal GC subtypes and subsequently performed gene set analysis for hallmark gene sets in the Molecular Signatures Database (MSigDB) [29] to determine overall functional differences. Using iMAT [13] and Metabolizer [14], we obtained metabolic subpathways (equivalent to GEM modules) involved in the two GC subtypes. To further validate the metabolic subpathway genes, we used independent transcriptome datasets and a metabolic profiling dataset (Figure 1).

### 3.2. Differential Expression between Diffuse and Intestinal GC Indicated Metabolic Context Differences

**Figure 2:**
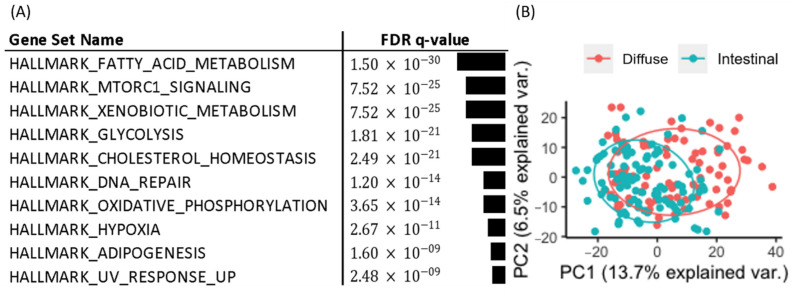
Hallmark gene set and PCA analysis for diffuse and intestinal GC subtypes in the TCGA. (**A**) Gene set analysis for DEGs of Lauren diffuse versus intestinal GC subtypes revealed metabolic contexts. (**B**) PCA of expression profiles of metabolism-related genes in diffuse and intestinal subtypes revealed separation between the two subtypes.

We obtained 537 DEGs (228 upregulated and 309 downregulated DEGs in diffuse versus intestinal GC subtypes) from 1379 metabolic genes from KEGG pathways with an FDR less than 0.1. To investigate how these DEGs affect metabolism in the diffuse and intestinal subtypes in TCGA patients with GC, we took the DEGs as input for the gene set enrichment analysis for hallmark gene sets in MIT MSigDB [17]. The top 10 statistically significant hallmark gene sets are shown in Figure 2A. Differential metabolism-related gene sets were also identified. Thus, we focused on metabolic pathway genes in the two GC subtypes in the following analyses.

We performed PCA based on the 1379 metabolic genes from KEGG pathways, and the results (Figure 2B) showed that the two subtypes were separated to some extent, indicating the existence of metabolic differences between the two subtypes.

### 3.3. iMAT Analysis Revealed Metabolic Reaction Differences between Diffuse and Intestinal GC Subtypes

**Figure 3:**
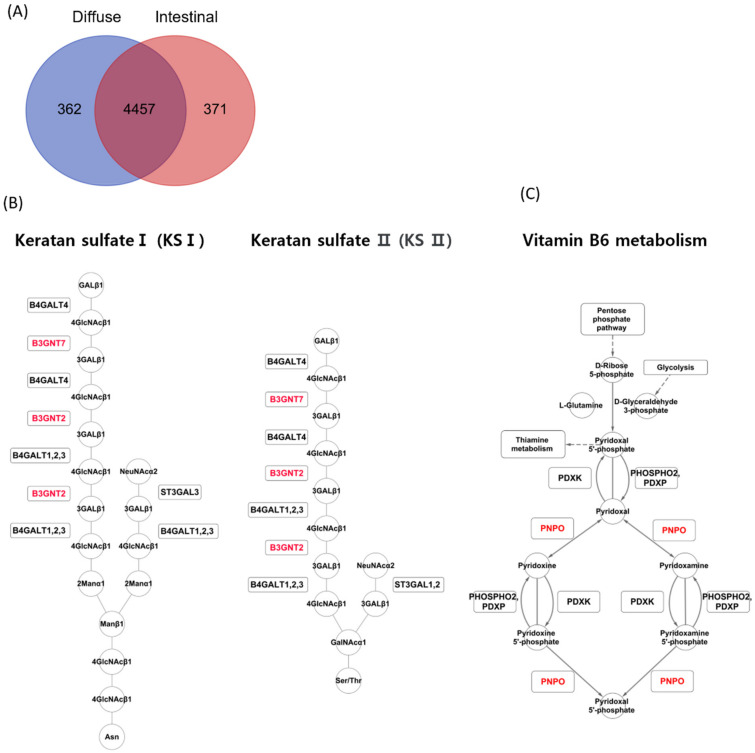
iMAT analysis for the two GC subtypes in the TCGA GC dataset. (**A**) Upregulated metabolic reactions in TCGA GC were obtained by iMAT for each subtype. (**B**) iMAT reported that *B3GNT7* and *B3GNT2* were involved in keratan sulfate synthesis in the diffuse subtype. (**C**) iMAT reported that *PNPO* was involved in vitamin B6 metabolism in the intestinal subtype.

The iMAT algorithm reported highly abundant reactions in diffuse and intestinal subtypes in TCGA patients with GC. In the diffuse subtype, 362 reactions were exclusively detected, while 371 reactions were detected exclusively in the intestinal subtype (Figure 3A). A reaction belonging to the keratan sulfate synthesis subsystem was detected exclusively in the diffuse subtype (Figure 3A). In contrast, reactions belonging to two subsystems, vitamin metabolism and dietary B6 binding, were detected exclusively in the intestinal subtype. iMAT reported that B3GNT7 and B3GNT2 were involved in keratan sulfate synthesis in the diffuse subtype (Figure 3B), and PNPO was involved in vitamin B6 metabolism in the intestinal subtype (Figure 3C).

### 3.4. Metabolizer Revealed Differential Activities between Diffuse and Intestinal Types

**Figure 4:**
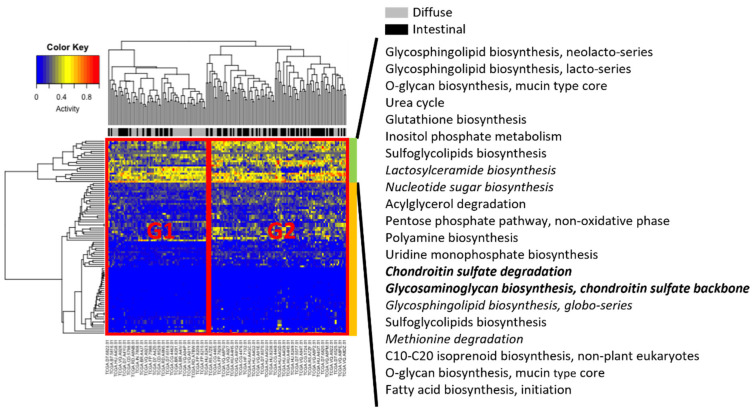
Metabolizer revealed differential activities between diffuse and intestinal subtypes in the TCGA. Columns indicate TCGA patients, and rows indicate activities of metabolic modules calculated by Metabolizer. The modules in italics indicate upregulation in diffuse versus intestinal subtypes, and the modules in italics and bold indicate statistically significant upregulation (adjusted *p* < 0.01).

For all samples, Metabolizer [14] was used to measure module activities, in which a module consists of sequential metabolic reactions in a subpart of a KEGG metabolic pathway (Figure 4). Two groups were associated with different clinical subtypes, indicated by G1 and G2 in Figure 4. Group G2 had more intestinal subtype patients than G1, whereas G1 had more diffuse subtype patients than G1. Visual inspection of hierarchical clustering revealed that Groups G1 and G2 were more differentiated in the upper part (indicated by the green column bar in Figure 4) than in the lower part (orange column bar in Figure 4). In particular, in the upper part, six modules (lactosylceramide biosynthesis; nucleotide sugar biosynthesis; chondroitin sulfate degradation; glycosaminoglycan biosynthesis, chondroitin sulfate backbone; glycosphingolipid biosynthesis, globo-series; and methionine degradation) were upregulated in diffuse compared to intestinal subtypes, and only two modules (chondroitin sulfate degradation; and glycosaminoglycan biosynthesis, chondroitin sulfate backbone) were significantly activated (adjusted *p* < 0.01).

### 3.5. Metabolizer Revealed Differential Metabolic Subpathways between Diffuse Versus Intestinal Types

**Figure 5:**
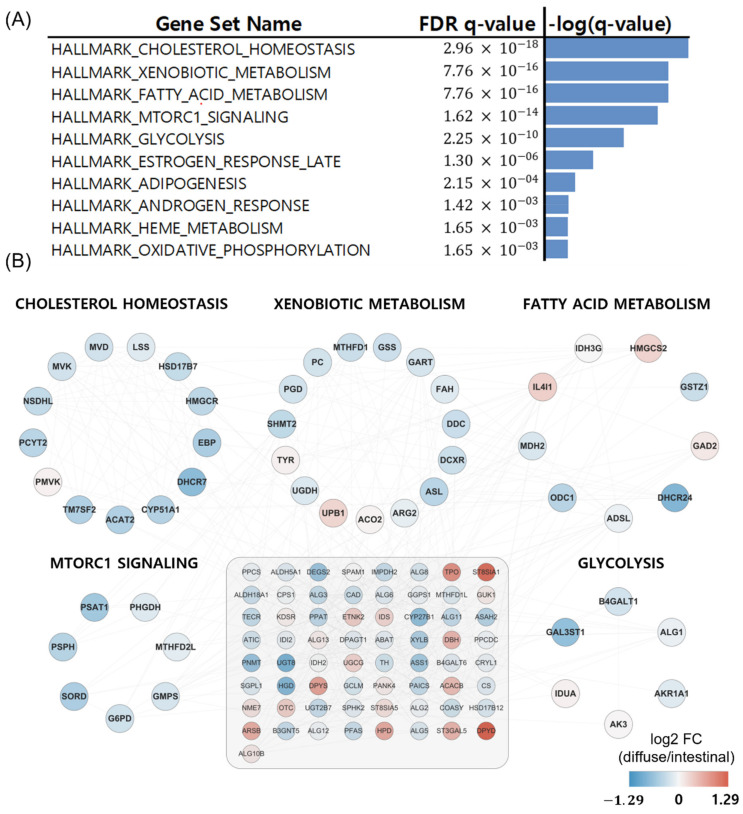
Metabolizer revealed differential metabolic subpathways in diffuse versus intestinal types in the TCGA. (**A**) Top ten significant pathways in diffuse versus intestinal subtypes. (**B**) Genes involved in the top five differential metabolic pathways. The 65 genes in the grey-shaded rectangle indicate the genes sharing the five differential metabolic pathways.

Metabolizer was used to identify additional metabolic contexts, including metabolic pathways associated with the diffuse and intestinal types. The top ten significantly enriched pathways in diffuse versus intestinal subtypes are summarized in Figure 5A, and five out of the ten are depicted in Figure 5B: cholesterol homeostasis, xenobiotic metabolism, fatty acid metabolism, MTORC1 pathway, and glycolysis. The five metabolic pathways had 49 genes involved in cancer [30,31,32,33]. Interestingly, these metabolic pathways were upregulated in patients with the intestinal subtype compared to those with the diffuse subtype (Figure 5B).

### 3.6. Validation of Significant Metabolic Pathways in Other GC Datasets and a Metabolic Profiling Dataset

**Figure 6:**
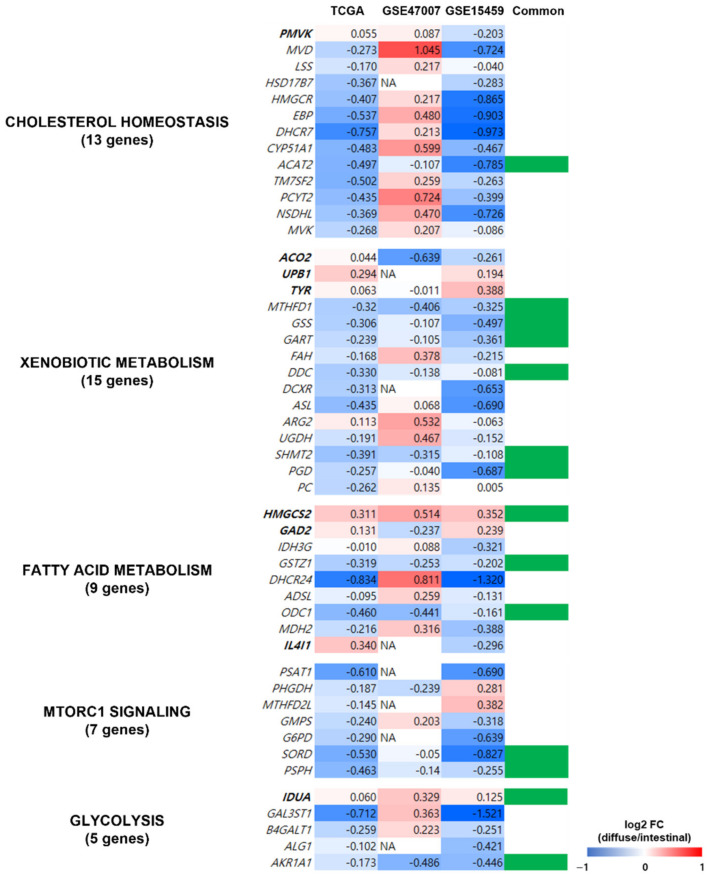
Validation of metabolism-related genes in the independent datasets of patients with GC. The expression profiles of the 49 genes of the five metabolic pathways (indicated in Figure 5B) were validated using gene expression profiles from two independent datasets of patients with GC (GSE15459 and GSE47007). Genes in bold were upregulated in diffuse compared to intestinal types in the TCGA GC dataset, and the genes in regular font were downregulated. Column “Common” indicates whether the gene showed similar gene expression profiles in all three datasets, and 14 genes (indicated in green in column Common) had similar expression profiles (diffuse versus intestinal subtypes) in TCGA and the other independent datasets.

To determine the reliability of the obtained metabolic pathways from the TCGA dataset, we validated the expression profiles of the 49 genes in five metabolic pathways (indicated in Figure 5B) using two independent datasets of patients with GC (GSE15459 [22] and GSE47007 [23]) and compared the pathways in diffuse versus intestinal GC subtypes. Out of the 49 genes, 14 (*ACAT2*, *MTHFD1*, *GSS*, *GART*, *DDC*, *SHMT2*, *PGD*, *HMGCS2*, *GSTZ1*, *ODC1*, *SORD*, *PSPH*, *IDUA*, and *AKR1A1*) showed similar gene expression profiles in the two independent datasets and the TCGA dataset (Figure 6). Thus, we confirmed that the pathways from the TCGA dataset were observed in other GC datasets.

In addition, we obtained gene expression profiles of diffuse and intestinal GC cell models. We reanalyzed gene expression profiles of the 49 genes in five metabolic pathways (indicated in Figure 5B) in diffuse (MKN1, KATOIII, and MKN45) and intestinal (MKN7 and NCI-N87) GC cell lines from a publicly available resource, the Genomics of Drug Sensitivity in Cancer (GDSC) [26]. Subsequently, we compared the 49 gene expression profiles of the TCGA dataset with those of the GC cell lines. As a result, 19 genes out of the 49 showed similar expression profiles in diffuse vs. intestinal GC subtypes throughout the five metabolic pathways (Figure S1). Thus, the results indicated that the pathways from the TCGA dataset were observed in the GC cell models.

We reanalyzed the dataset of metabolites in diffuse and intestinal GC cell lines to identify differential metabolites [27,34]. As a result, statistical tests indicated that C18-carnitines, oleylcarnitine and stearoylcarnitine, were statistically significantly abundant in diffuse versus intestinal subtypes (*p* < 0.05) (Figure S2). Additionally, C16-caritine, palmitoylcarnitine, was statistically marginally abundant in diffuse versus intestinal subtypes (*p* = 0.063) (Figure S2). Recently, these lipid species were closely associated with fatty acid metabolism [28,35], which agrees with our finding of lower expression of fatty acid metabolism genes in diffuse versus intestinal subtypes (Figure 5B). This is further described in the Discussion section.

## 4. Discussion

Metabolism in GC has received little attention thus far, while much effort has been devoted to the identification of signaling pathways in GC [36,37,38]. In this study, we examined the metabolic differences between the diffuse and intestinal GC subtypes using two methods: iMAT [13] and Metabolizer [14]. iMAT [13] extracts group-specific (context-specific) models from GEMs [10,39], which represent all metabolic reactions in a cell. Both methods obtain a subset of GEMs by eliminating inactive reactions based on transcriptomics [10]. To the best of our knowledge, this is the first report of the metabolic differences between Lauren GC subtypes that utilized transcriptomics-based GEMs.

In the iMAT analysis (Figure 3), keratan sulfate and vitamin B6 metabolism were associated with the diffuse and intestinal GC subtypes, respectively. Keratan sulfate is a glycosaminoglycan [40] that is covalently attached to protein cores to form proteoglycans and plays important roles in cancer cell migration, angiogenesis, and epithelial-to-mesenchymal transition [41,42]. Keratan sulfate may contribute to carcinogenesis in diffuse-type GC, but its role in diffuse-type GC needs further study.

During the conversion of dietary vitamin B6 to pyridoxal 5′-phosphate (PLP), the physiologically active form of vitamin B6, PNPO (pyridox (am) ine 5′-phosphate oxidase), is a rate-limiting enzyme [43]. PNPO and vitamin B6 metabolism are associated with cancer [44,45]. It has been shown that vitamin B6-deficient cancer cells are more resistant to apoptosis than vitamin B6-proficient lung cancer cells [45,46]. The diffuse GC subtype is less sensitive to chemotherapeutics than the intestinal GC subtype [24]. Considering that vitamin B6 metabolism is highly activated in the intestinal GC subtype (Figure 3), vitamin B6 metabolism could be involved in the different chemotherapy responses between the diffuse and intestinal subtypes, which warrants further investigation in the future.

Chondroitin sulfate degradation was significantly enriched in the diffuse GC subtype compared to intestinal GC subtypes (Figure 4). Chondroitin sulfate is a glycosaminoglycan covalently bound to protein cores that produce proteoglycans [47]. Cancer cell–associated chondroitin sulfates appear to promote the migration and invasion of cancer cells; however, there are exceptions [47]. For example, certain chondroitin sulfates (e.g., chondroitin-6-sulfates) inhibit the migration and invasion of B16V melanoma cells [48]. Due to the context-dependent function of chondroitin sulfates on the migration and invasion of cancer cells, the role of chondroitin sulfate degradation (Figure 4) in the diffuse GC subtype affects the malignancy of the subtype.

In Figure 5, we visualize networks generated by Metabolizer to observe the interactions among the entries. Cholesterol homeostasis, fatty acid metabolism, the MTORC1 pathway, glycolysis, and xenobiotic metabolism were upregulated in the intestinal subtype compared to the diffuse subtype [30,31,32,33]. Interestingly, cholesterol homeostasis, fatty acid metabolism, the MTORC1 pathway, and glycolysis are involved in lipid homeostasis [49], implying that carcinogenesis of the intestinal subtype is more likely to be associated with lipid homeostasis than the diffuse subtype.

Cholesterol homeostasis, as well as cholesterol itself, is associated with well-known oncogenic pathways [50]. In the mevalonate pathway of cholesterol homeostasis, the production of farnesyl pyrophosphate and geranylgeranyl pyrophosphate induces the prenylation of oncoproteins, small Ras family GTPases, and their downstream effectors [50]. In addition, activation of LXR during cholesterol homeostasis induces an anti-proliferative effect in GC cells [50,51].

In xenobiotic metabolism (Figure 5), cytochrome P450 enzymes (CYP450s) play an important role in bridging the environment and the body [32]. CYP450s have pleiotropic effects on cancer [32]. For example, they can activate xenobiotics into carcinogens and metabolize prodrugs into active drugs [32]. In addition, altered expression of CYP450s often confers drug resistance and increased proliferation to cancer cells [32]. However, the mechanism by which differential xenobiotic metabolic activity between diffuse and intestinal subtypes affects drug responses and carcinogenesis is still unknown [52].

Cancer cells often utilize fatty acids as energy sources via β-oxidation during metabolic stress conditions [31]. In addition, fatty acid metabolism is closely related to tumor microenvironments, because fatty acids can be provided as energy sources to cancer cells by adipocytes [53,54]. Tumor growth in adipocyte-rich environments has often been observed in diverse cancer types, including GC [55]. However, despite the strong association between cancer and fatty acid metabolism, the activation of fatty acid metabolism in GC subtypes has yet to be experimentally validated.

Cancer cells utilize anaerobic glycolysis to generate insufficient adenosine triphosphate (ATP) but produce massive intermediates necessary for cell proliferation [56]. As shown in Figure 5, glycolysis was upregulated in the intestinal subtype compared to the diffuse subtype. Based on PET/CT scans that revealed cell glucose metabolism using ^18^F-2-fluoro-2-deoxy-D-glucose (^18^F-FDG) as a tracer, less FDG uptake was observed in the diffuse subtype (compared with the intestinal subtype), which depends on GLUT-1 expression [56]. This is partly due to the low expression of glucose transporter 1 (GLUT1) in the diffuse subtype [56].

The MTORC1 pathway (i.e., mTOR pathway) was upregulated in intestinal compared to diffuse subtypes. This is consistent with the upregulation of fatty acid metabolism and glycolysis in intestinal versus diffuse subtypes (Figure 5). This is partly because PI3K–AKT–mTOR signaling promotes glucose consumption via glycolysis to confer evolutionary advantages to cancer cells [31,56,57]. The mTOR pathway is related to fatty acid metabolism because MTORC1 regulates lipogenesis through the activation of SREBP1 (sterol regulatory element-binding protein 1) and SRPK2 (SR-protein-specific kinase 2), thereby inducing the expression of lipogenic enzymes, including ACLY (ATP–citrate lyase), FASN (fatty acid synthase), and ACSS2 (acyl-CoA synthetase short-chain family member 2) [31,56]. The benefit of sustaining the lipogenesis of fatty acids is the flexibility to shunt fatty acids into diverse biosynthetic pathways to produce various cellular pools of lipid species with distinct functions [31].

Fourteen of the forty-nine genes associated with the five metabolic pathways (cholesterol homeostasis, fatty acid metabolism, MTORC1 pathway, glycolysis, and xenobiotic metabolism) obtained in the networks (Figure 5B) were replicated in the two datasets of patients with GC (Figure 6), indicating the reliability of the metabolic pathways. A thorough literature search revealed that at least 7 of the 14 genes were associated with poor prognosis and cancer cell proliferation in cancer, including *ACAT2*, *GART*, *SHMT2*, *HMGCS2*, *GSTZ1*, *PSPH*, and *IDUA*. Therefore, the five metabolic pathways (Figure 5B) should be further investigated. Downregulation of *ACAT2* is associated with poor prognosis in clear cell renal cell carcinoma [58]. High expression of *GART* in hepatocellular carcinoma (HCC) is associated with poor prognosis and promotes cancer cell proliferation [59]. High *SHMT2* expression is associated with poor prognosis and lymphatic invasion in GC [60]. Low *HMGCS2* expression enhances the proliferation and metastasis of HCC [61]. Downregulation of *GSTZ1* was observed in HCC, which also indicated a poor prognosis [62]. High *PSPH* expression is associated with poor prognosis in HCC [63]. *IDUA* was included in a risk score model to evaluate survival risk in an ovarian cancer patient [64].

In the lipid metabolite analysis (Figure S2), long-chain (LC) carnitine species, oleylcarnitine, stearoylcarnitine, and palmitoylcarnitine, were elevated in intestinal versus diffuse subtypes. Recently, a fatty acid oxidation (β-oxidation) decrease was associated with the elevation of C14, C16, and C18 LC acylcarnitine species, including oleylcarnitine, stearoylcarnitine, and palmitoylcarnitine [28]. LC fatty acids acting as energy sources in cancer are esterified with carnitine to LC acylcarnitine species in the cytoplasm [65,66,67], and LC acylcarnitine species are transported and degraded in mitochondria for fatty acid oxidation, generating ATPs [68,69]. Thus, a decrease in fatty acid oxidation may be associated with the accumulation of LC acylcarnitine species. This fact might indicate that, in our study, fatty acid metabolism via β-oxidation was lower in diffuse versus intestinal subtypes (Figure 5B). Further experimental validation is awaited.

The use of transcriptome-based GEMs to investigate metabolic pathways has a limitation: the concentrations of metabolites were not considered in our study. Since our study utilized bioinformatics analyses on public datasets, the results should be carefully interpreted. In the future, metabolite profiling of tissues in patients with GC should be performed to accurately describe metabolic differences between GC subtypes.

## 5. Conclusions

In this study, we applied GEMs to identify differences in metabolic pathways between the diffuse and intestinal GC subtypes. We utilized transcriptome-based GEMs, and the results require further validation through metabolomics or metabolite analysis. Moreover, how these metabolic subpathways affect the different prognoses between diffuse and intestinal subtypes needs to be further investigated.

## Data Availability

Publicly available datasets were analyzed in this study. These data can be found here: UCSC Xena [15], GEO accessions GSE15459 [22] and GSE47007 [23], DepMap [27], and GDSC [26].

## Supplementary Materials

The following supporting information can be downloaded at: https://www.mdpi.com/article/10.3390/cancers14092340/s1, Figure S1: Validation of the metabolism-related genes in an independent dataset of diffuse and intestinal GC cell lines; Figure S2: Lipid abundance differences between diffuse and intestinal GC cell lines, by re-analyzing the experiments of hydrophilic interaction chromatography and reversed phase chromatography in DepMap.
