# Supplementary material for: Genome-Scale Metabolic Model Analysis of Metabolic Differences between Lauren Diffuse and Intestinal Subtypes in Gastric Cancer
Source: Cancers (Basel). 2022 May 9;14(9):2340. doi: 10.3390/cancers14092340 (PMC9104812; doi:10.3390/cancers14092340)
Supplement: Supplementary file 1 [file cancers-14-02340-s001.zip › cancers-1713524-supplementary.pdf]

Supplementary Materials: Genome-Scale Metabolic Model Analysis of Metabolic Differences between Lauren Diffuse and Intestinal Subtypes in Gastric Cancer

Seungyoon Nam and Yongmin Lee

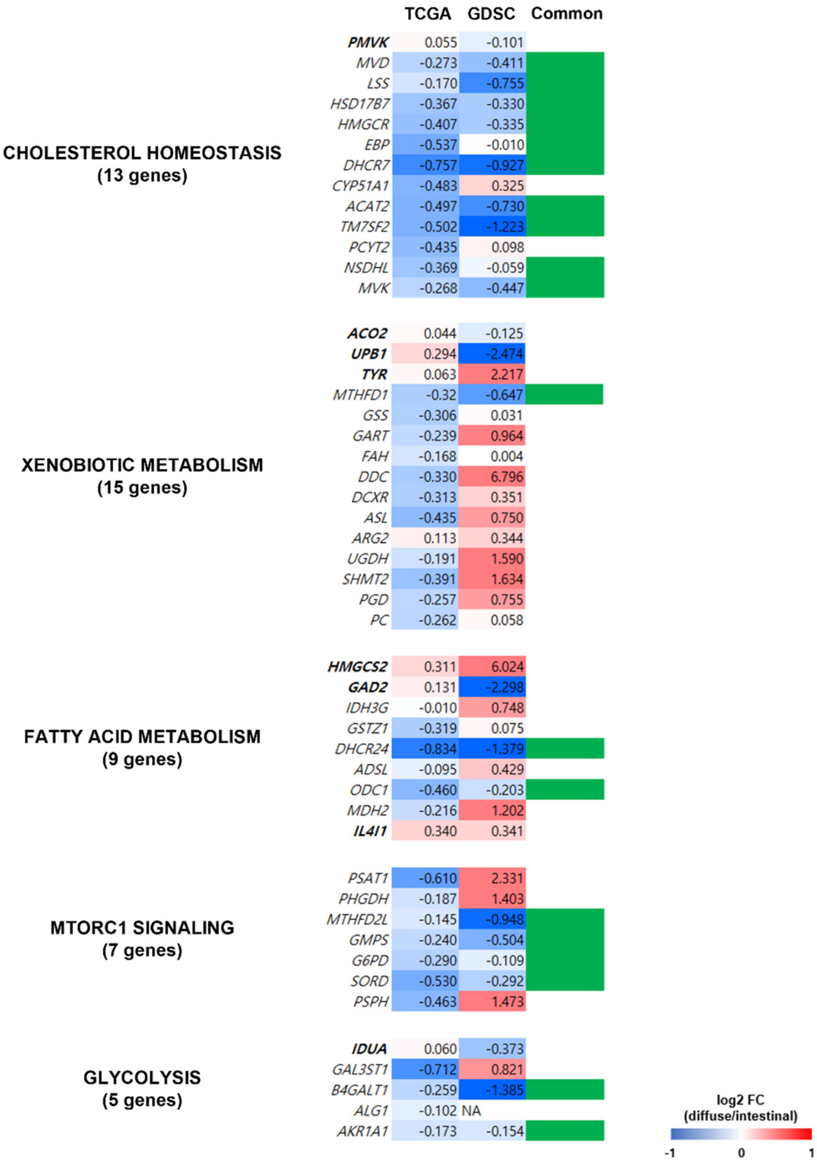

Figure S1. Validation of the metabolism-related genes in an independent dataset of diffuse and intestinal GC cell lines. The expression profiles of the 49 genes of the five metabolic pathways (indicated in Fig. 5B) were validated using gene expression profiles of another independent dataset of diffuse and intestinal GC cell lines from GDSC [1]. Genes in bold were upregulated in diffuse compared intestinal types in the TCGA GC dataset, and the genes in regular font were downregulated. Column "Common" indicates whether the gene showed similar gene expression profiles in the two datasets (TCGA and GDSC). Nineteen genes (indicated in green in column Common) throughout the five metabolic pathways (i.e., cholesterol homeostasis, xenobiotic metabolism, fatty acid metabolism, MTORC1 signaling and glycolysis) had similar expression profiles (diffuse over intestinal subtypes) between the GC cell lines and TCGA patients with GC.

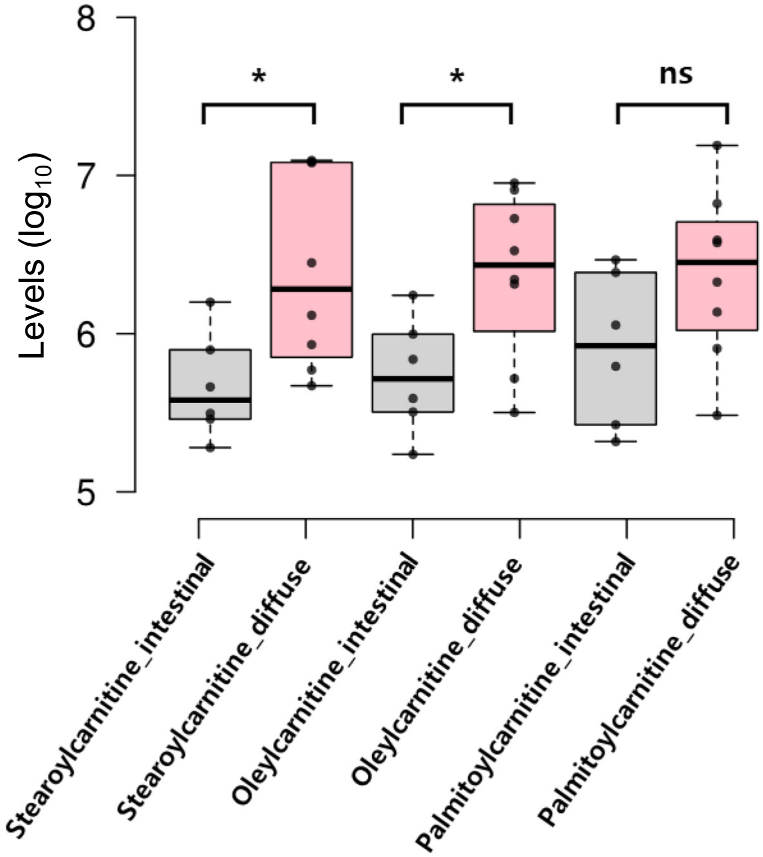

Figure S2. Lipid abundance differences between diffuse and intestinal GC cell lines, by re-analyzing the experiments of hydrophilic interaction chromatography and reversed phase chromatography in DepMap. The C18-carnitines, oleylcarnitine and stearoylcarnitine, were statistically significantly abundant in diffuse over intestinal subtypes ($p < 0.05$). The C16-caritine, palmitoylcarnitine, was statistically marginally abundant in diffuse over intestinal subtypes (p=0.063). *: $p < 0.05$; ns:non-significant.

Reference

1. Yang, W.; Soares, J.; Greninger, P.; Edelman, E.J.; Lightfoot, H.; Forbes, S.; Bindal, N.; Beare, D.; Smith, J.A.; Thompson, I.R.; et al. Genomics of Drug Sensitivity in Cancer (GDSC): a resource for therapeutic biomarker discovery in cancer cells. *Nucleic Acids Res.* **2013**, *41*, D95–-D961, doi:10.1093/nar/gks1111.
